# The Pseudoknot Region of the 5′ Untranslated Region Is a Determinant of Viral Tropism and Virulence of Foot-and-Mouth Disease Virus

**DOI:** 10.1128/JVI.02039-18

**Published:** 2019-04-03

**Authors:** Zixiang Zhu, Fan Yang, Weijun Cao, Huanan Liu, Keshan Zhang, Hong Tian, Wen Dang, Jijun He, Jianhong Guo, Xiangtao Liu, Haixue Zheng

**Affiliations:** a State Key Laboratory of Veterinary Etiological Biology, National Foot and Mouth Diseases Reference Laboratory, Key Laboratory of Animal Virology of Ministry of Agriculture, Lanzhou Veterinary Research Institute, Chinese Academy of Agricultural Sciences, Lanzhou, China; Instituto de Biotecnologia/UNAM

**Keywords:** foot-and-mouth disease virus, host range, pathogenicity, pseudoknots

## Abstract

Foot-and-mouth disease virus (FMDV) is the causative agent of foot-and-mouth disease. It is characterized by genetic instability and different antigenic properties. The nonstructural protein 3A is a primary determinant of the tropism and virulence of Cathay topotype FMDVs. However, several other determinants are also speculated to be involved in viral tropism and virulence. Deletion of 43 nucleotides (nt) in the pseudoknot (PK) region of the 5′ untranslated region (UTR) has been found to coexist with the identified 3A deletion in Cathay topotype FMDV genomes. In this study, we isolated an O/ME-SA/PanAsia lineage FMDV strain, O/GD/CHA/2015, that includes an 86-nt deletion in the PK region and shows a porcinophilic phenotype. To investigate the potential role of the PK region in viral pathogenicity, we generated a recombinant FMDV strain with an incomplete PK region and compared its virulence and pathogenesis to the intact FMDV strain in swine and bovines. Deletion of the 86 nt in the PKs had no major effects on the pathogenicity of the virus in swine but significantly attenuated its ability to infect bovine cells and cattle, indicating that the PK region is a newly discovered determinant of viral tropism and virulence. The role of the 43-nt deletion existing in the Cathay topotype FMDV was also investigated by evaluating the infection properties of genetically engineered viruses. Consistently, the 43-nt deletion in the PK region significantly decreased the pathogenicity of the virus in bovines. Overall, our findings suggest that the PK region deletion occurred naturally in the FMDV genome and that the PK region is highly associated with viral host range and functions as a novel determinant for FMDV pathogenesis.

**IMPORTANCE** This study demonstrates that the deletion in the PK region occurred naturally in the FMDV genome. The isolated O/ME-SA/PanAsia lineage FMDV with an 86-nt deletion in the PK region showed a pig-adapted characteristic that could cause clinical signs in swine but not bovines. Compared to the wild-type FMDV strain, which possesses full infection capacity in both swine and bovines, the recombinant virus with the 86-nt deletion in the PK region is deficient in causing disease in bovines. Deletion of the previously reported 43 nt in the PK region also led to significantly decreased pathogenicity of FMDV in bovines. This study indicates that the PK region is a novel determinant of the tropism and virulence of FMDV.

## Abstract (precis)

This study demonstrates that the deletion in the PK region occurred naturally in the FMDV genome. The isolated O/ME-SA/PanAsia lineage FMDV with an 86-nt deletion in the PK region showed a pig-adapted characteristic that could cause clinical signs in swine but not bovines. Compared to the wild-type FMDV strain, which possesses full infection capacity in both swine and bovines, the recombinant virus with the 86-nt deletion in the PK region is deficient in causing disease in bovines. Deletion of the previously reported 43 nt in the PK region also led to significantly decreased pathogenicity of FMDV in bovines. This study indicates that the PK region is a novel determinant of the tropism and virulence of FMDV.

## INTRODUCTION

Foot-and-mouth disease (FMD) is a highly contagious viral disease caused by foot-and-mouth disease virus (FMDV). Outbreaks of FMD often result in severe economic losses due to its effect on trade and the slaughtering of infected animals. The wide host range and high degree of contagiousness makes FMD difficult to control and eradicate (1). FMDV belongs to the genus *Aphthovirus* in the family *Picornaviridae* and consists of seven serotypes (O, A, C, SAT1, SAT2, SAT3, and Asia l) and multiple subtypes within each serotype (2, 3). Three serotypes of FMDV, including serotypes O, A, and Asia 1, have caused epidemics in Asia that make FMD difficult to control, and serotype O FMDVs are mainly responsible for current outbreaks of FMD in Asian countries (4, 5).

FMDV is an RNA virus characterized by high genetic variation and antigenic diversity, and cross-serotype protection is not always developed among different FMDV populations (6). In addition, in some regions, viral infection with one genetic lineage does not confer full protection against another genetic lineage in the same serotype. Different genetic lineages of the same serotype that fall within different geographical boundaries are defined as different topotypes, and different topotypes have evolved independently (7). Serotype O is the most prevalent of the seven serotypes of FMDV and is considered one of the most antigenically diverse serotypes (8, 9). FMDV serotype O is classified into 11 topotypes based on phylogenetic analysis of the VP1 coding region, including Europe–South America (Euro-SA), Middle East–South Asia (ME-SA), Southeast Asia (SEA), Cathay (CHY), West Africa (WA), East Africa 1 (EA-1), East Africa 2 (EA-2), East Africa 3 (EA-3), East Africa 4 (EA-4), Indonesia-1 (ISA-1), and Indonesia-2 (ISA-2) (8–10). Rapid evolution in nature results in the long-term survival and spread of FMDV throughout a wide region and makes FMD difficult to eradicate.

The FMDV 5′ untranslated region (UTR) plays an important role during FMDV replication. The 5′ UTR is >1,300 bp long and contains a short segment known as the S fragment, which follows the poly(C) tract, the pseudoknots (PKs), the *cis*-acting replication element (*cre*), and the internal ribosome entry site (IRES) (11). The PK region is predicted to be involved in the conserved structures of the FMDV genome (12). However, the role of the PK region remains largely unknown (13). It is believed that the PK region may be involved in viral RNA replication (14). Nucleotide sequence deletions have been observed in the PK region in FMDV O, A, and C serotypes, which raises interesting possibilities about the hitherto-unknown evolutionary function of this region. Previous studies have identified a 10-amino-acid deletion in the 3A protein that is responsible for the decreased pathogenicity of Cathay topotype FMDVs in bovines. A 43-nucleotide (nt) deletion in the PK region of the 5′ UTR of Cathay topotype FMDV that coexists with the 3A deletion was also observed (15). However, the role of this deletion in the PK region remains unclear. We isolated an O/ME-SA/PanAsia lineage FMDV, strain O/GD/CHA/2015, that contains an intact 3A protein and an 86-nt deletion in the PK region of the 5′ UTR. The role of the 86-nt deletion in the PKs also remains unclear. O/GD/CHA/2015 showed a pig-adapted characteristic: that the virus could cause clinical signs in swine but not in bovines. To investigate the potential function of the PK region, we evaluated the association of the PK region with viral pathogenicity. Using a reverse genetics system, we demonstrated that the 86 nt in the PK region are an important determinant of host range for O/ME-SA/PanAsia lineage FMDVs. We also demonstrated that artificial deletion of 43 nt that naturally occurs in the PK region of the porcinophilic Cathay topotype FMDV genome resulted in decreased pathogenicity of the O/ME-SA/PanAsia lineage FMDVs in bovines. Our results show a novel role of the PK region of FMDV as a host range determinant and imply that natural deletions in the PK region of serotype O FMDVs might cause a possible shift from cattle to swine as the carrier for the spread of FMDV.

## RESULTS

### Isolation of a new O/ME-SA/PanAsia lineage FMDV strain.

**FIG 1:**
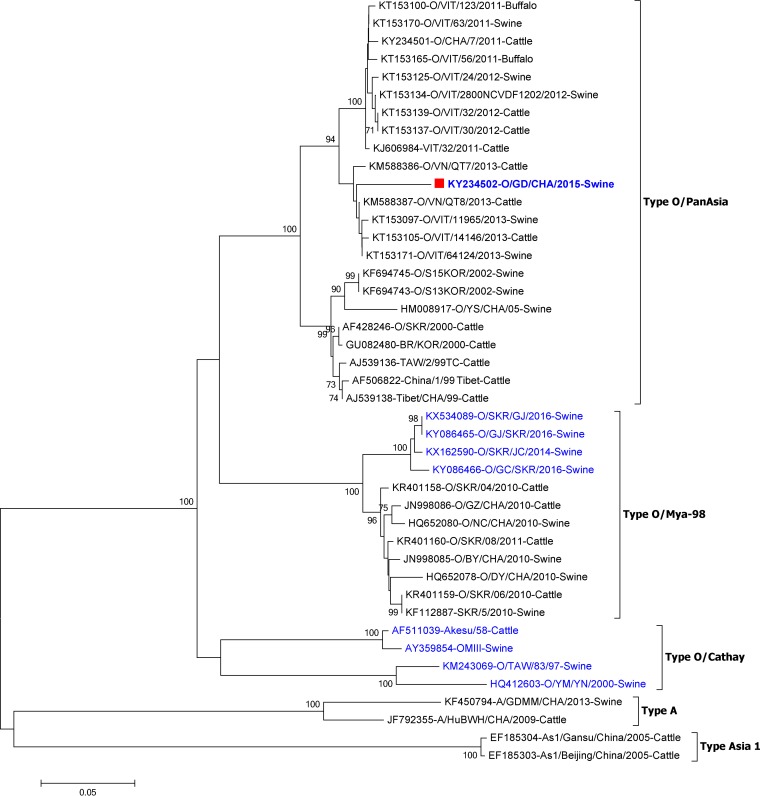
The FMDV O/GD/CHA/2015 strain belongs to the O/ME-SA/PanAsia lineage. Phylogenetic analysis of the VP1 sequences of FMDV O/GD/CHA/2015 and the other 42 FMDV isolates was carried out. ClustalX 1.83 and MEGA 6.06 software were used for construction of the tree. The analyses were carried out using the neighbor-joining method and the Kimura two-parameter nucleotide substitution model in MEGA 6.06 (www.megasoftware.net). The number of bootstrap replicates was set at 1,000. All other parameters were default values. Partial PK region deletions found in the viral genome of the FMDV strains are marked in blue.

A new serotype O FMDV strain was isolated from swine in the Guangdong province of China in 2015. The isolated virus strain was named O/GD/CHA/2015. The complete genome of O/GD/CHA/2015 was subsequently sequenced and analyzed to investigate its molecular characteristics. Analysis of FMDV population variation has been performed mainly by aligning the VP1 gene (1). Therefore, the VP1 nucleotide sequence of the O/GD/CHA/2015 strain was first compared to the nucleotide database in GenBank using BLAST (NCBI). The VP1 gene of O/GD/CHA/2015 shared the highest sequence identity (96%) with a type O strain, O/VN/QT8/2013, isolated from bovines in Vietnam in 2013, which belongs to the PanAsia lineage O/ME-SA topotype (O/ME-SA/PanAsia). The VP1 sequences of 42 other representative isolates belonging to different serotypes or topotypes from China and other Asian countries were used for phylogenetic analysis. The O/GD/CHA/2015 strain shares a closer phylogenetic relationship with the recent O/ME-SA/PanAsia lineage strains from Vietnam than with contemporary O/SEA/Mya-98 lineage FMDV strains from China (Fig. 1). This confirmed that O/GD/CHA/2015 belongs to the PanAsia lineage O/ME-SA topotype (O/ME-SA/PanAsia).

### O/GD/CHA/2015 contains an 86-nt deletion in the PKs of the 5′ UTR, and all the porcinophilic Cathay topotype FMDVs also contain partial deletions in the PK region.

**FIG 2:**
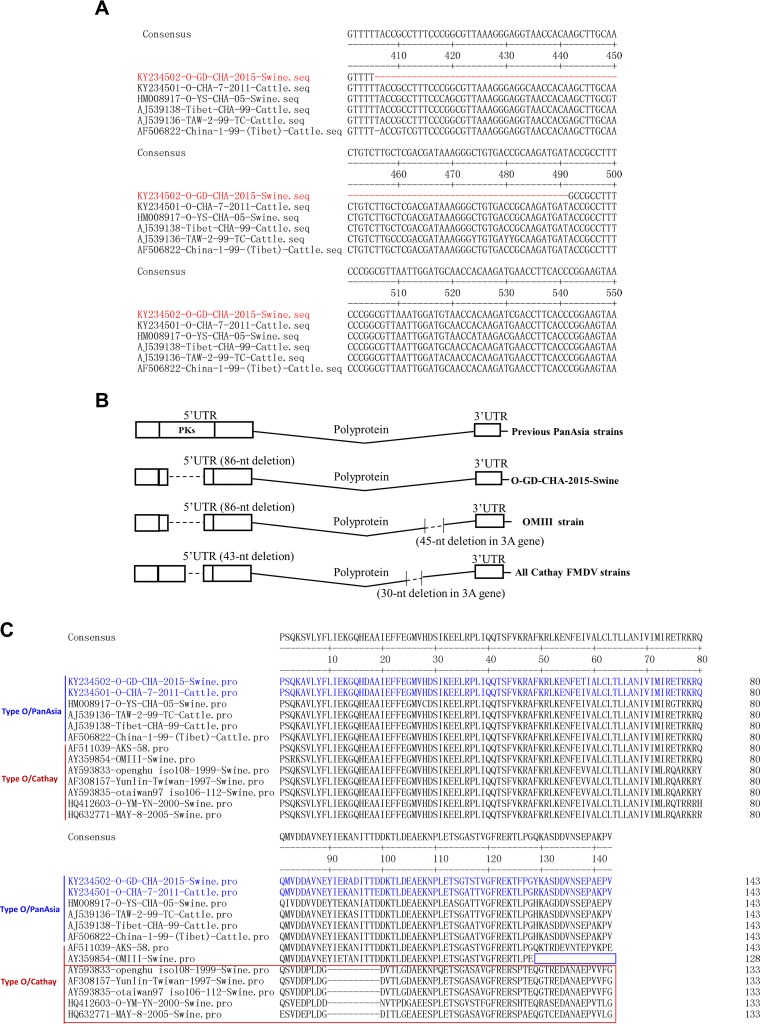
A PK region deletion in the serotype O FMDV genome occurred naturally in pigs, and all the porcinophilic Cathay topotype FMDVs also included a partial PK region deletion. (A) The 86-nt deletion in the PK region identified in O/GD/CHA/2015 after comparison to other PanAsia FMDV strains reported previously in China. The red lines indicate the deleted region in O/GD/CHA/2015. (B) Schematic representation showing the 86-nt deletion in the PK region of O/GD/CHA/2015 compared to 70 other published serotype O FMDVs isolated in Asian countries available in the NCBI database. (C) Comparison of the 3A protein sequences of Cathay topotype FMDV strains, O/GD/CHA/2015, and the other O/ME-SA/PanAsia lineage viruses isolated in China. The red box indicates the previously reported porcinophilic Cathay topotype FMDV strains with 10 amino acids in the 3A protein. The blue box indicates a 15-amino-acid deletion in the 3A protein of the FMDV OMIII strain. The sequences of 3A proteins of O/GD/CHA/2015 and O/CHA/7/2011 are marked in blue.

Comparison of the viral genome of O/GD/CHA/2015 with other O/ME-SA/PanAsia lineage viruses in China was performed. Nucleotide sequence analysis of the genome revealed that O/GD/CHA/2015 differed from the previous O/ME-SA/PanAsia lineage viruses isolated in China (83.6 to 94.8% identity) and was most closely related to the O/CHA/7/2011 strain. A deletion of 86 nt was observed within the PKs of the 5′ UTR of O/GD/CHA/2015 at positions 406 to 491, compared to other O/ME-SA/PanAsia FMDV strains (Fig. 2A). As for polyprotein comparison, all of these O/ME-SA/PanAsia lineage viruses contained 2,332 amino acids (no deletions or insertions were observed in O/GD/CHA/2015), and O/GD/CHA/2015 also showed the highest amino acid identity with the O/CHA/7/2011 strain (97.8%). The 3′ UTR of O/GD/CHA/2015 was the same length as that of O/CHA/7/2011 and also shared the highest nucleotide identity (91.6%). To investigate the stability of the 86-nt deletion in the PK RNA region, the 5′ UTR sequences of the isolate and the fourth-passage progeny virus in BHK-21 cells were determined and compared. The results showed that the progeny virus stably holds the 86-nt deletion in the 5′ UTR region (data not shown). O/GD/CHA/2015 included a considerably different 5′ UTR from that of the other O/ME-SA/PanAsia lineage strains. To further investigate the characteristics of the 5′ UTR of O/GD/CHA/2015, 70 published 5′ UTR sequences of serotype O FMDVs isolated in Asian countries (including different topotypes) available in the NCBI database (with detailed isolation information in GenBank) were subsequently extracted and compared using DNA STAR software. We found that all the Cathay topotype FMDVs also harbored deletions in the PK region of the 5′ UTR (Fig. 2B). A 10-amino-acid deletion was identified in the 3A protein of the porcinophilic Cathay topotype FMDVs, as previously reported (16) (Fig. 2C), and these porcinophilic FMDVs, concurrently harbored 43-nt deletions in the PK region of the 5′ UTR. A previously isolated Chinese Cathay topotype of serotype O virus OMIII (an artificially attenuated virus from strain Akesu/58) contained an 86-nt deletion in the PK region similar to that in the O/GD/CHA/2015 strain (Fig. 2B) and included a 15-amino-acid deletion in 3A (Fig. 2C). O/GD/CHA/2015 and other O/ME-SA/PanAsia lineage viruses all included intact 3A protein (Fig. 2C). O/GD/CHA/2015 is not a Cathay topotype strain, and it has an intact 3A protein; however, it also includes a partial PK region deletion. The role of these deletions in the PK region remains unknown.

### O/GD/CHA/2015 shows a pig-adapted characteristic.

Previous studies reported that the 10-amino-acid deletion in the 3A protein of serotype O Cathay topotype virus strain O/TAW/97 resulted in an altered host tropism (pig adapted) and a severe epidemic among pig farms (16). Moreover, evidence suggests that some other viral determinants might also exist and are associated with host range specificity (17, 18). The OMIII strain is a pig-adapted Cathay topotype strain. All the porcinophilic Cathay topotype FMDV strains include partial PK region deletions. O/GD/CHA/2015 included an 86-nt deletion in the PK region and had an intact 3A protein. This prompted us to figure out whether the PK region deletion resulted in a similar porcinophilic phenotype to that caused by the 3A deletion in the Cathay topotype FMDV strains. To investigate this hypothesis, the host range of O/GD/CHA/2015 was subsequently investigated. In parallel, a Chinese O/ME-SA/PanAsia lineage strain, O/CHA/7/2011, with an intact PK region was used as a control during all the animal experiments. O/CHA/7/2011 included the complete PK region in the 5′ UTR (Fig. 2A). Alignment of the polyprotein and 3′ UTR sequences of O/CHA/7/2011 and O/GD/CHA/2015 was performed. Both O/CHA/7/2011 and O/GD/CHA/2015 included intact 3A protein and polyprotein, and no insertion or deletion was observed in the 3′ UTR (data not shown). Therefore, O/CHA/7/2011 was used as the control. Different species have different challenge routes during FMDV inoculation. Intradermal inoculation is the most certain method of producing infection by FMDV in cattle (19). Intramuscular inoculation of FMDV can cause generalized disease in pigs (5, 20). Therefore, FMDV challenge by intradermal injection in cattle and intramuscular injection in pigs has been recommended by both China and the World Organisation for Animal Health (OIE) as preferred methods for FMDV challenge experiments (16, 19–23). Groups of five swine or cattle that were serologically negative by FMDV enzyme-linked immunosorbent assay were used for virus challenge. The pigs were challenged by intramuscular injection with 10^7^ 50% tissue culture infective doses (TCID_50_)/animal of either FMDV. This dose had previously been shown to be a high dose for FMDV that can cause clinical disease in swine (24, 25). Doses of 10^7^ and 10^8^ TCID_50_/animal of either FMDV were used to infect cattle by intradermal inoculation. The dose of 10^8^ TCID_50_/animal was a high dose that caused clinical disease during the experiments. All the animals were monitored daily for clinical signs of disease after viral challenge. Clinical scores were determined by the following criteria as previously described (26). Briefly, the mouth, nostril, or tongue lesion beyond the inoculation site was assigned a score of 1 and one or more lesions per foot a score of 1. The maximum score was 5.

**FIG 3:**
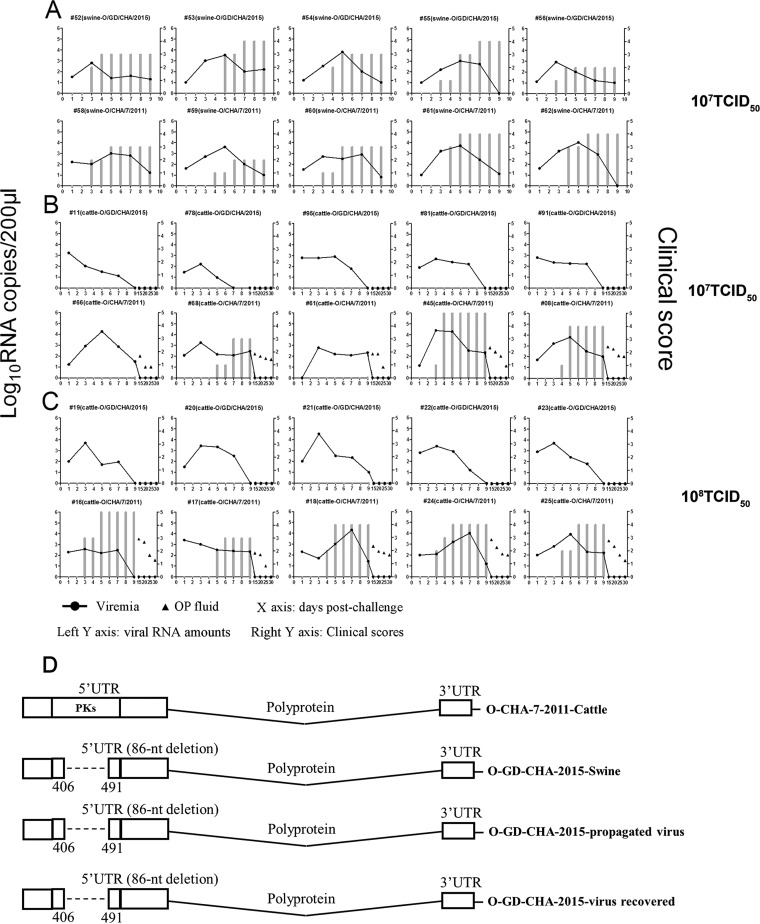
O/GD/CHA/2015 had no pathogenic ability in cattle. (A) Ten pigs were challenged by intramuscular injection with 10^7^ TCID_50_/animal of O/GD/CHA/2015 (with a deletion in the PK region) or O/CHA/7/2011 (with the complete PK region). Clinical signs were monitored daily and viremia was determined at 1, 3, 5, 7, and 9 dpc. (B and C) The cattle were challenged by intradermal inoculation with 10^7^ TCID_50_/animal (B) or 10^8^ TCID_50_/animal (C) of O/GD/CHA/2015 or O/CHA/7/2011. Clinical signs were monitored daily and viremia was determined at 1, 3, 5, 7, 9, 15, 20, 25, and 30 dpc. Viral RNAs in the bovine OPF collected at 15, 20, 25, and 30 dpc were measured using qPCR. The left *y* axes represent the viral RNA amounts (viremia and OP fluid), and the right *y* axes represent the clinical scores (the gray bar graphs indicate the recorded clinical scores at different days postchallenge). The *x* axes represent the day postchallenge. (D) Comparison of the 5′ UTR region sequences of O/GD/CHA/2015 obtained from the tissue specimens, the propagated O/GD/CHA/2015 in BHK-21 cells, the O/GD/CHA/2015 recovered from the challenged pigs, and the O/CHA/7/2011 strain (a schematic representation).

Both the O/CHA/7/2011 and O/GD/CHA/2015 strains affected pigs, and all the pigs showed significant clinical disease (severe lesions at 3 days postchallenge [dpc]) and viremia (Fig. 3A). For the cattle, regardless of whether the low or high doses of virus were used in the challenge, only O/CHA/7/2011 caused clinical disease. Two cattle (animals 66 and 61) challenged with 10^7^ TCID_50_/animal of O/CHA/7/2011 showed no visible lesions during the experiments, possibly due to unavoidable logistical and operational constraints or because this dose is insufficient to cause severe disease in all cattle (Fig. 3B). However, all the cattle challenged with the high dose of 10^8^ TCID_50_/animal of O/CHA/7/2011 showed significant clinical lesions (Fig. 3C). In addition, the virus was detected in the oropharyngeal fluid (OPF) from all the cattle challenged with O/CHA/7/2011 at 15, 20, and 25 dpc (Fig. 3B and C). In contrast, O/GD/CHA/2015 failed to cause any clinical disease even at the high dose of inoculum. Viral RNA was undetectable in the OPF throughout the whole experiment, and no viruses were isolated from the OPF and blood. The viremia disappeared faster than in the cattle challenged with O/CHA/7/2011 (Fig. 3B and C). We could recover O/GD/CHA/2015 from challenged pigs but not from cattle. These data suggested that the O/GD/CHA/2015 strain has significantly decreased pathogenicity in cattle, but is swine susceptible. This implies that the PK region might be a novel determinant that is implicated in the host range of O/ME-SA/PanAsia lineage FMDVs. Meanwhile, no reversion or partial reversion was observed in the 5′ UTR of virus recovered from challenged pigs (Fig. 3D). The other genomic regions of the viruses were also sequenced. No additional mutations were observed in the viral proteins or noncoding regions (data not shown). This suggested that the 86-nt deletion in the PK region was stable during viral challenge experiments.

### Construction of two recombinant viruses with or without the 86-nt deletion in the PK region.

**FIG 4:**
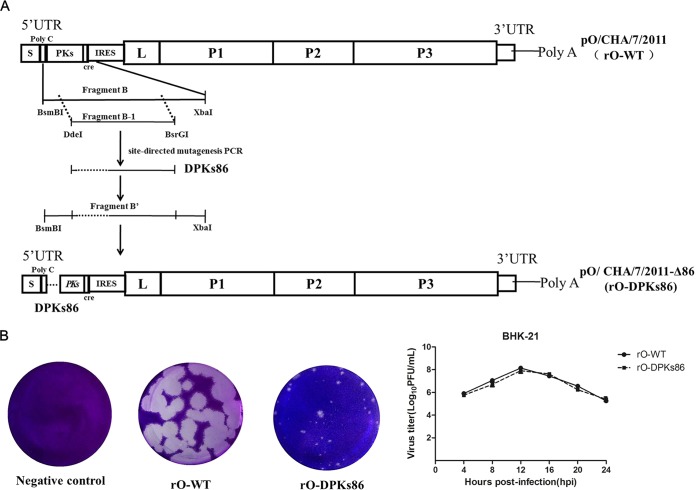
Construction and rescue of two recombinant viruses, rO-WT and rO-DPKs86. (A) Schematic representation showing the constructs of pO/CHA/7/2011 and pO/CHA/7/2011-Δ86. (B) Plaque assays of rO-WT and rO-DPKs86 on BHK-21 cells and the determined virus titers at the indicated time points.

To further determine the potential effect of the 86-nt deletion within the PK region of O/ME-SA/PanAsia lineage virus on its pathogenicity, two recombinant viruses based on the PanAsia virus O/CHA/7/2011 were generated using a reverse genetics system. We constructed a plasmid, pO/CHA/7/2011, and a derivative of plasmid pO/CHA/7/2011 (pO/CHA/7/2011-Δ86) containing an 86-nt deletion in the PK region. Transfection of the pO/CHA/7/2011 plasmid in BHK-21 cells generated a wild-type O/CHA/7/2011 FMDV with a full-length PK region (rO-WT), while transfection of pO/CHA/7/2011-Δ86 generated a mutant O/CHA/7/2011 strain with an 86-nt deletion in the PK region (rO-DPKs86) (Fig. 4A). After four consecutive passages in BHK-21 cells, an indirect immunofluorescence assay was performed to identify the rescued viruses. BHK-21 cells infected with the rescued viruses were immunostained by polyclonal antibodies specific for serotype O FMDV VP3 and exhibited green fluorescence (data not shown). The plaque-forming assay revealed that both viruses caused visible cytopathic effect (CPE) in BHK-21 cells (Fig. 4B). Unexpectedly, rO-DPKs86 had a smaller plaque size than rO-WT; however, the two viruses had almost identical virus yields (Fig. 4B). These data demonstrated the successful rescue of rO-WT and rO-DPKs86 viruses by reverse genetics. The PK regions of the two viruses were subsequently sequenced to confirm the designed distinction of the rescued viruses (86-nt difference in the PK region). Amplification of the PK region showed that an 86-nt deletion was observed in rO-DPKs86; however, no deletions were detected in the rO-WT. Other regions of the two viral genomes were further sequenced to confirm that, apart from the 86-nt deletion, the sequences of rO-WT and rO-DPKs86 were identical. The results indicated that, apart from the 86-nt deletion, some synonymous substitutions were found in the polyprotein-coding sequence. However, no amino acid deletion or insertion was observed, which suggested that there was no difference between rO-WT and rO-DPKs86 viral proteins and noncoding regions except for the 86-nt in the PK region.

### The 86-nt deletion in the PKs contributed to decreased pathogenicity of O/CHA/7/2011 in bovine cells and cattle.

**FIG 5:**
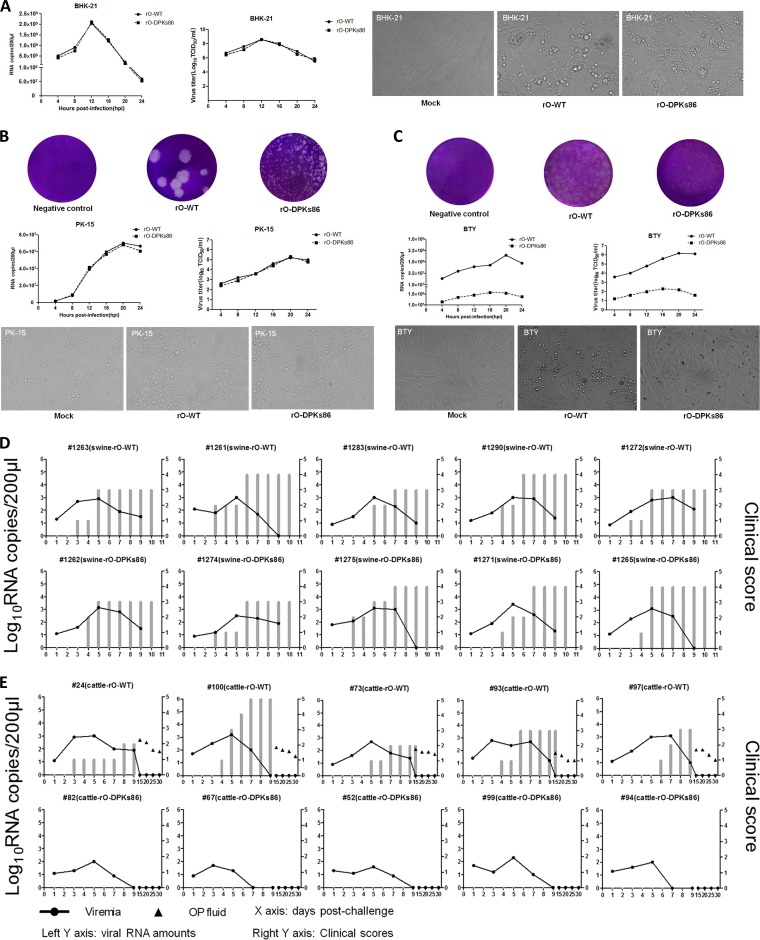
rO-DPKs86 showed significantly decreased infective ability for bovine cells and cattle compared with rO-WT. (A) Amounts of viral RNA in rO-WT- or rO-DPKs86-infected BHK-21 cells at 4, 8, 12, 16, 20, and 24 hpi (left panel); one-step growth curves for rO-WT and rO-DPKs86 in BHK-21 cells (middle panel); viral CPE caused by rO-WT and rO-DPKs86 in BHK-21 cells at 16 hpi (right panel). (B) Plaque morphology caused by rO-WT or rO-DPKs86 in PK-15 cells (upper panel); viral RNA levels and virus loads in rO-WT- or rO-DPKs86-infected PK-15 cells at 4, 8, 12, 16, 20, and 24 hpi (middle panel); viral CPE caused by rO-WT and rO-DPKs86 in PK-15 cells at 20 hpi (lower panel). (C) Plaque morphology caused by rO-WT or rO-DPKs86 in BTY cells (upper panel); viral RNA levels and virus loads in rO-WT- or rO-DPKs86-infected BTY cells at 4, 8, 12, 16, 20, and 24 hpi (middle panel); viral CPE caused by rO-WT and rO-DPKs86 in BTY cells at 24 hpi (lower panel). (D) Ten pigs were challenged by intramuscular injection with 10^7^ TCID_50_/animal of rO-WT or rO-DPKs86. Clinical signs were monitored daily and viremia was determined at 1, 3, 5, 7, and 9 dpc. The left *y* axes represent the viral RNA levels (log_10_RNA copies/200 μl), and the right *y* axes represent the clinical scores (the gray bar graphs indicate the recorded clinical scores at different days postchallenge). The *x* axes represent the day postchallenge. (E) Ten cattle were challenged by intradermal inoculation with 10^8^ TCID_50_/animal of rO-WT or rO-DPKs86. Clinical signs were monitored daily, and viremia was determined at 1, 3, 5, 7, 9, 15, 20, 25, and 30 dpc. Viral RNAs in the bovine OPF collected at 15, 20, 25, and 30 dpc were measured.

After successful rescue of the two recombinant viruses, the growth kinetics of rO-WT and rO-DPKs86 were compared in BHK-21 cells, a permissive cell line for FMDV propagation. Despite that rO-DPKs86 formed smaller plaques than rO-WT in BHK-21 cells (Fig. 4B), there were no differences in viral RNA replication and particle production between rO-DPKs86 and rO-WT (Fig. 4B and 5A). The ability of the viruses to form large or small plaques did not correlate with the ability to replicate in the growth curve experiment, which was similar to previous observations for influenza A viruses (27). In addition, the two viruses caused similar extents of visible CPE in BHK-21 cells (Fig. 5A). To explore whether the 86-nt deletion in the PK region was a genetic determinant involved in the variation of the host tropism of the virus from cattle to swine, bovine-derived BTY and swine-derived PK-15 cells were used to evaluate the replication of rO-WT and rO-DPKs86 *in vitro*. The results showed that both viruses are heterogeneous in plaque sizes but homogeneous in RNA replication, viral yield, and CPE extent (Fig. 5B). Interestingly, although rO-WT and rO-DPKs86 revealed same-sized plaques in BTY cells, rO-WT had a significant growth advantage over rO-DPKs86, and rO-WT caused a more dramatic CPE than rO-DPKs86 (Fig. 5C). This indicates that the 86-nt deletion in the PK region decreases viral replication in BTY cells but not in BHK-21 or PK-15 cells.

To compare the replication and pathogenicity of the rescued viruses *in vivo*, a parallel challenge experiment similar to that shown in Fig. 3 was performed. Equal amounts of rO-WT or rO-DPKs86 (doses of 10^7^ TCID_50_/pig and 10^8^ TCID_50_/cattle) were used to infect cattle or pigs. All the challenged animals were monitored daily for clinical signs of disease after inoculation of the viruses. Both rO-WT and rO-DPKs86 resulted in clear clinical disease and viremia in the challenged pigs (Fig. 5D). However, in the cattle, rO-DPKs86 only caused slight viremia, and no clinical signs of FMD were observed compared to rO-WT. Viruses could be detected in the OPF from the rO-WT-infected cattle at the indicated times, but not in the OPF from the rO-DPKs86-infected cattle (Fig. 5E). We could recover rO-DPKs86 from the challenged pigs but not from cattle. The results of the *in vitro* and *in vivo* experiments indicated that the 86-nt deletion in the PK region conferred an altered virulence of the virus in cattle. The 5′ UTR sequences of the viruses used for viral challenge and the viruses recovered from the challenged pigs were also determined and compared. No reversion or partial reversion was observed in the 5′ UTR region of rO-DPKs86. Other genomic regions of the viruses were also sequenced. The results indicated that there were some synonymous substitutions in the polyprotein-coding sequences. However, no amino acid deletion or insertion was observed, which suggested that no additional mutation occurred in the viral proteins. In addition, no mutations were observed in the noncoding regions. This further suggested that the 86-nt deletion in the PK region was stable during viral challenge experiments.

### Deletion of the 43 nt that were naturally lost in the Cathay topotype FMDVs also significantly decreased the pathogenicity of O/CHA/7/2011.

**FIG 6:**
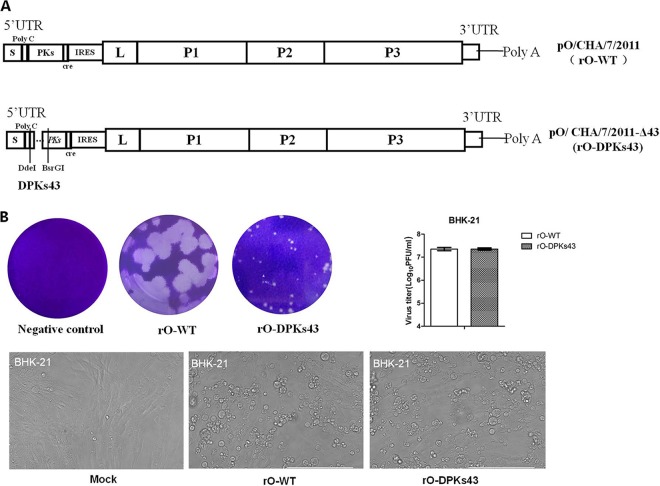

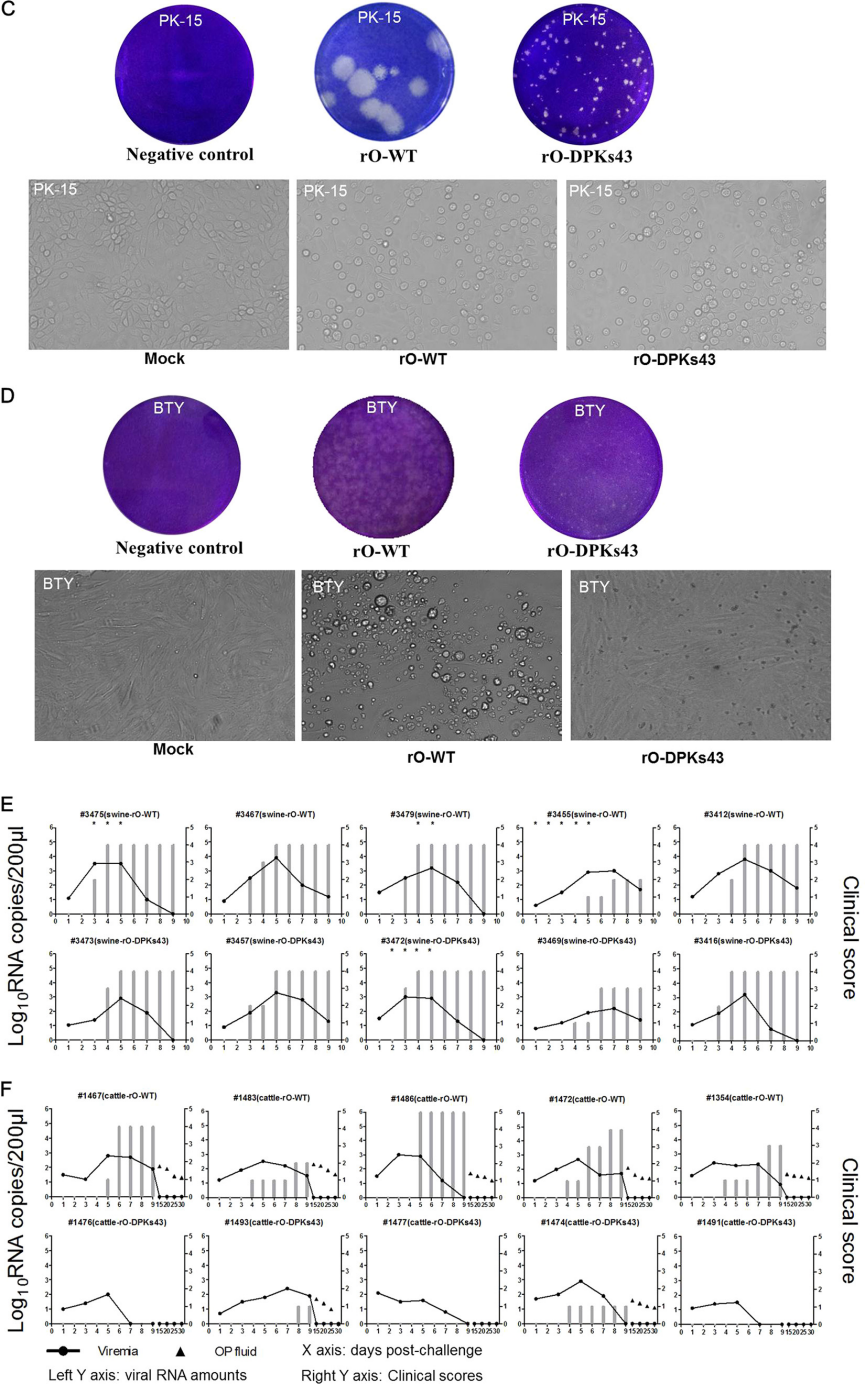
rO-DPKs43 showed significantly decreased infective ability in cattle compared to rO-WT. (A) Structures of genetically engineered virus genomes of rO-WT and rO-DPKs43. (B) Plaque assays, virus titers, and the CPE observation of rO-WT and rO-DPKs43 in BHK-21 cells. (C) Plaque morphology and CPE caused by rO-WT or rO-DPKs43 in PK-15 cells. (D) Plaque morphology and CPE caused by rO-WT or rO-DPKs43 in BTY cells. (E) Ten pigs were challenged by intramuscular injection with 10^7^ TCID_50_/animal of rO-WT or rO-DPKs43. Clinical signs were monitored daily, and viremia was determined at 1, 3, 5, 7, and 9 dpc. The left *y* axes represent the viral RNA levels (log_10_RNA copies/200 μl), and the right *y* axes represent the clinical scores (the gray bar graphs indicate the recorded clinical scores at different days postchallenge). The *x* axes represent the day postchallenge. (F) Ten cattle were challenged by intradermal inoculation with 10^8^ TCID_50_/animal of rO-WT or rO-DPKs43. Clinical signs were monitored daily and viremia was determined at 1, 3, 5, 7, 9, 15, 20, 25, and 30 dpc. Viral RNAs in the bovine OPF collected at 15, 20, 25, and 30 dpc were measured.

Apart from the Chinese Cathay topotype virus OMIII that harbored an 86-nt deletion in the PK region similar to that in O/GD/CHA/2015, all the porcinophilic Cathay topotype FMDVs harbored a 43-nt deletion in the PK region of the 5′ UTR. The role of the 43 nt in the PK region was further investigated by constructing a recombinant virus, rO-DPKs43, using a reverse genetics system and performing similar animal experiments, as mentioned above. A derivative of plasmid pO/CHA/7/2011 containing a 43-nt deletion in the PK region (pO/CHA/7/2011-Δ43) was constructed by replacing part of the 5′ UTR region (Fig. 6A). Transfection of pO/CHA/7/2011-Δ43 into BHK-21 cells also successfully produced the recombinant virus rO-DPKs43. The ability of the viruses to form large or small plaques also did not correlate with the ability to replicate in the growth curve experiment in BHK-21 cells, and the two viruses caused similar extents of visible CPE (Fig. 6B). Amplification of the PK region showed that 43 nt were deleted in rO-DPKs43. Other regions of rO-DPKs43 were sequenced, which indicated that, apart from the 43-nt deletion, there was no difference between the viral elements of rO-WT and rO-DPKs43. Plaque morphology and CPE extent in PK-15 and BTY cells were also assessed for the rescued viruses. The results showed that rO-DPKs43 also formed smaller plaques than rO-WT in PK-15 cells; however, the two viruses caused similar extents of visible CPE (Fig. 6C). In the BTY cells, rO-WT and rO-DPKs43 revealed similar plaque sizes, but rO-WT caused a more dramatic CPE than rO-DPKs43 (Fig. 6D). This indicates that the 43-nt deletion in the PK region decreases viral replication in BTY cells but not in BHK-21 or PK-15 cells.

To compare the replication and pathogenicity of the rescued viruses *in vivo*, a parallel animal challenge experiment was also performed. Five pigs or five cattle were challenged with equal amounts of rO-WT or rO-DPKs43 (10^7^ TCID_50_/pig and 10^8^ TCID_50_/cattle), and clinical signs were monitored daily. rO-WT and rO-DPKs43 caused disease in pigs, revealing similar pathogenicities (Fig. 6E). However, rO-DPKs43 resulted in significantly decreased pathogenicity in cattle compared to rO-WT. All the cattle challenged with rO-WT showed severe lesions, and viral RNA was detected from all the OPF samples. In contrast, only two of five cattle challenged with rO-DPKs43 showed moderate or mild lesions, with positive viral RNA detection in the OPF samples, and the other three cattle showed no clinical signs, and their OPF samples were also viral RNA negative (Fig. 6F). This indicates that the 43-nt deletion in the PK region resulted in decreased virulence of the O/ME-SA/PanAsia lineage viruses in cattle. This 43-nt deletion in the PK region in Cathy topotype FMDVs might be another genetic determinant, apart from those reported previously, that is responsible for the porcinophilic properties of Cathy topotype viruses. The 5′ UTR sequences of the viruses used for viral challenge and the viruses recovered from the challenged pigs were determined and compared. No reversion or partial reversion was observed in the 5′ UTR region of rO-DPKs43. Other genomic regions of the viruses were also sequenced. No additional mutations were observed in the viral proteins or noncoding regions. This suggested that the 43-nt deletion in the PK RNA region was stable during viral challenge experiments.

## DISCUSSION

Three serotypes of FMDV, including serotypes O, A, and Asia 1, have caused epidemics in Asian countries that make FMD difficult to control, and serotype O is the most prevalent of the three serotypes of FMDV and has occurred in many regions in Asia. In recent years, serotype O FMDVs have been mainly responsible for outbreaks of FMD in China (28). The present study describes a novel pig-adapted serotype O FMDV in China that was determined to be an O/ME-SA/PanAsia lineage strain with several undiscovered characteristics.

It has been reported that the O/ME-SA/PanAsia lineage is not restricted to infection of pigs (16, 29). However, we identified a pig-adapted O/ME-SA/PanAsia lineage virus, O/GD/CHA/2015, that caused disease in pigs but not in cattle. A deletion in the PK region of the 5′ UTR at position 406 to 491 was observed in O/GD/CHA/2015 compared to previous O/ME-SA/PanAsia lineage FMDVs. The role of this 86-nt deletion in viral pathogenicity was further studied by construction of recombinant viruses. rO-DPKs86 had smaller average plaques than rO-WT in BHK-21 cells, while the two viruses showed similar replication rates. The ability of rO-WT and rO-DPKs86 to form large or small plaques did not correlate with the ability to replicate in the growth curve experiment in BHK-21 cells. Plaque size is highly dependent on the use of certain viral mutants, strains, and cell types, and it cannot be fully considered a reliable marker for viral virulence, leaving the study of this crucial biological feature to the realm of animal experimentation (30–35). For strains of some viruses, there is a connection between the replication rate and virulence (35, 36). Previous studies also observed that several viruses and their mutant viruses could form different plaque sizes but present similar virus replication profiles (27, 37). For FMDV, it has been suggested that the plaque phenotype is not necessarily of significance for production of FMDV vaccine antigen (38). Here, our data also showed that the ability of rO-WT and rO-DPKs86 to form large or small plaques did not correlate with the ability of the viruses to replicate in BHK-21, PK-15, and BTY cells. This suggests that plaque size does not significantly affect the replication of FMDV in these cells. We have also observed this phenomenon in our previous studies when comparing the plaque sizes of some different FMDV recombined viruses to their wild-type viruses. Some wild-type viruses can cause large plaque sizes, but their mutants do not. We speculate that these wild-type viruses may cause fusion of infected cells with their neighboring cells, while mutation of some regions of the viral genome may change this characteristic. In the animal experiments, we found that this 86-nt deletion in the PK region significantly contributed to the altered virulence of the virus in cattle. The rescued virus with the deletion showed pathogenicity in swine but not in cattle. Low levels of viremia were detected in the rO-DPKs86-challenged cattle, but no clinical signs were observed, no viral loads were detected from the target tissues of the virus, and viremia disappeared quickly in the cattle. This implied that rO-DPKs86 might have lost infectivity in cattle. These results indicated that the 86-nt region at positions 406 to 491 within the PK region of FMDV is a potential determinant of host tropism variation, and under some circumstances altered PK regions might have a selective advantage. FMDV in animals evolves under immune pressure and reveals new characteristics. The accumulated mutations may help the viruses better adapt themselves to the environment.

China is a densely pig-populated country. In recent years, the serotype O FMDVs identified in China were isolated mainly from pigs, and there has been a decreasing incidence of FMD caused by serotype O viruses in cattle (28, 39). This is possibly because the serotype O viruses have circulated in pigs for a long time and some of the viruses have evolved to adapt to pigs and thus had more chance to replicate in pigs. The large number of pigs can better promote the spread of the virus. Therefore, the altered host tropism (pig adapted) might be beneficial to the spread of FMDVs. The deletions in the PK region of the virus imply that some of the O/ME-SA/PanAsia lineage viruses have evolved a predilection to affect pigs, and a possible shift from cattle to swine as the carrier for the spread of O/ME-SA/PanAsia lineage FMDVs might be under way in China. In addition, we observed that four O/SEA/Mya-98 lineage viruses isolated in South Korea in 2014 and 2016 also included a similar deletion in the PK region (data not shown). The four strains were all isolated from pigs, and the host tropism characteristics of these FMDV strains should be further investigated to explore the role of the PK region in O/SEA/Mya-98 lineage viruses.

Previous studies have reported that the deletion in the 3A protein of serotype O Cathay topotype virus strain O/TAW/97 at amino acids 93 to 102 resulted in altered host tropism (pig adapted) and severe epidemics among swine farms (17). The virus harboring this deletion showed significantly attenuated infective ability in both bovine cells and cattle. The deletion in the O/TAW/97 strain is similar to one in the egg-passaged derivatives of FMDV (O1 Campos) that also show reduced ability to affect cattle (16). This indicates that under some circumstances altered 3A genes could have a selective advantage. In this study, we found that all the porcinophilic Cathay topotype FMDVs also harbored a 43-nt deletion in the PK region of the 5′ UTR, apart from a previously identified Chinese Cathay topotype of serotype O virus OMIII that harbored an 86-nt deletion in the PK region similar to that in O/GD/CHA/2015. After comparison of the viral pathogenicity of rO-DPKs43 and rO-WT in swine and cattle, we determined that the deletion of the 43 nt significantly decreased the virulence of O/CHA/7/2011 in cattle. OMIII strain is a pig-adapted Cathay topotype strain with a similar 86-nt deletion in the PK region that significantly affects only swine and does not cause disease in cattle. These data imply that the PK region might also be a determinant involved in the variation of the host tropism of Cathay topotype viruses.

The Cathay topotype FMDVs were sampled mainly in southeast and east Asian countries (8, 40). The economic losses associated with an invasion of a pig-adapted FMDV strain of the Cathay topotype of serotype O in Taiwan in 1997 have been estimated at 6 billion U.S. dollars, with the slaughtering of >4 million pigs (41). The first Cathay topotype FMDV strain was isolated from pigs in Hong Kong SAR in 1970. Subsequently, Cathay strains have been isolated from many Asian and several European countries (40, 42). Cathay topotype FMDVs have been continually maintained within the swine industry close to Hong Kong SAR from 1995 to 2005, following the extinction of virus lineages from the Philippines and a reduced number of FMD cases in Taiwan, according to a reconstructed phylodynamics analysis, and the Cathay strains have been sampled on a more sporadic basis in recent years (40). The 10-amino-acid deletion in the 3A protein has been shown to correlate with the inability of previous Cathay topotype FMDVs to cause disease in bovines (16, 17). The mutations in Cathay topotype viruses have accumulated over several decades, and the deletions in the PK region might be another determinant. The deletions in the PK region and 3A protein of Cathay topotype viruses may cooperate and result in the inability of Cathay topotype FMDVs to cause disease in bovines.

The function of the PK region of FMDV remains unknown. In this study, molecular epidemiological research and viral pathogenicity evaluation allowed us to hypothesize that the 86 nt within the PK region of the 5′ UTR were related to the host tropism variation of serotype O FMDVs. Using rescue of recombinant viruses and animal experiments, we reported the first evidence supporting the hypothesis that the 86-nt deletion in the PK region occurred naturally and resulted in decreased pathogenicity of O/ME-SA/PanAsia lineage virus in bovine cells and in cattle. In addition, we deduced that the 43-nt deletion in the PK region of porcinophilic Cathay topotype FMDVs might be another genetic determinant that is responsible for its porcinophilic properties. This study identified the PK region as a pathogenic determinant of FMDVs that was associated with host tropism variation, and the generated data will contribute to exploring the functions of the PK region of FMDVs during viral infection.

## MATERIALS AND METHODS

### Ethics statements.

All animal experiments were approved and performed according to the requirements of the Gansu Animal Experiments Inspectorate and the Gansu Ethical Review Committee [license SYXK(GAN) 2010-003].

### Viruses and cells.

Baby hamster kidney (BHK-21) cells and porcine kidney (PK-15) cells were cultured as described previously (43). Primary bovine thyroid (BTY) cells were maintained in medium containing 10% fetal bovine serum. All the cells were cultured at 37°C under 5% CO_2_. The O/GD/CHA/2015 strain was isolated from swine tissue in Guangdong Province, China. The O/CHA/7/2011 strain was isolated from bovine oropharyngeal fluid (OPF) in Guizhou Province, China. We determined the genomic sequences of the O/GD/CHA/2015 and O/CHA/7/2011 strains (accession numbers KY234502 and KY234501, respectively).

### Genetic and phylogenetic analyses.

The GenBank accession numbers and information on the FMDV isolates used for phylogenetic tree construction are listed in Fig. 1. Phylogenetic analysis was performed on aligned subgenomic regions of FMDV utilizing ClustalX 1.83, the neighbor-joining method, and the Kimura two-parameter nucleotide substitution model in MEGA6.06 software (www.megasoftware.net) as previously described (44). One thousand bootstrap replicates were performed in the analysis. The nucleotide sequences of viral 5′ UTR were aligned using the MegAlign project of Lasergene software (Lasergene, Madison, WI).

### Construction of rO-WT, rO-DPKs86, and rO-DPKs43 infectious clones.

Viral RNA was extracted from O/CHA/7/2011 FMDV-infected BHK-21 cells for use as the template for cDNA synthesis. The strategy for construction of the plasmid used to produce the recombinant virus was as described previously by our laboratory (45). The viral genome was amplified using three pairs of primers, and the PK deletion was introduced into the second fragment (fragment B) by site-directed mutagenesis PCR. Briefly, fragment B was inserted into the pOK12 vector. DdeI and BsrGI digestion enzymes were used to obtain the fragment B-1, and the 86- or 43-nt deletions were then introduced into the fragment B-1. Fragment B with the 86- or 43-nt deletion was named fragment B′. The first and the third fragments and fragment B or fragment B′ was then cloned into the pcDNAP_1_T_1_ vector. The full-length viral genome or the genome with PK deletions was cloned into the pcDNAP_1_T_1_ vector, and the purified plasmids were sequenced.

### Viral rescue and identification.

The highly purified pO/CHA/7/2011, pO/CHA/7/2011-Δ86, and pO/CHA/7/2011-Δ43 plasmids were obtained using MN NucleoBond Xtra Midi Plus plasmid DNA purification kits (Macherey-Nagel, Duren, Germany) according to the manufacturer’s protocol. The extracted plasmid DNA was transfected into BHK-21 cells (about 2 μg/10^6^ cells) using Lipofectamine 2000 (Invitrogen, Carlsbad, CA) for 48 h. The culture supernatant was harvested and blind passaged into secondary BHK-21 cells. After four consecutive passages in BHK-21 cells, the rescued viruses were detected and analyzed using an immunofluorescence assay and a plaque titration assay on BHK-21 cells. The primer set of Del-F (5′-CCCCCCCCCCCCCCAAGTTTT-3′) and Del-R (5′-CCTTCTCAGATCCCGAGTGTCG-3′) was used to amplify the PK regions of the rescued viruses to confirm successful deletion of the 86 or 43 nt. The viral genomes of the obtained rescued viruses were further sequenced using the Sanger sequencing method.

### Determination of the replication kinetics of the rescued viruses.

The replication kinetics of rO-WT, rO-DPKs86, and rO-DPKs43 were determined by analyzing the one-step growth curve of viral titers through a TCID_50_ assay (46). Equal doses of rO-WT, rO-DPKs86, or rO-DPKs43 were inoculated into monolayer cells. The infected cells were collected at various time points and subjected to virus load titration using their corresponding cells. The results are representative of three independent experiments.

### Animal experiments.

The World Organisation for Animal Health (OIE) recommends that FMDV research be conducted in biosafety level 4 (BSL4) laboratories, and this is generally applicable to FMD-free countries. In other countries and regions, the virus is approved to be handled in BSL3 laboratories (47). Therefore, all the animal experiments were performed in a confined environment and high-containment facilities (animal BSL3) of the Lanzhou Veterinary Research Institute according to the standard method of the OIE. In the first experiment, 10 pigs (age, 10 weeks; weight, ∼40 kg) or 10 cattle (age, 7 months; weight, ∼100 kg) were divided into two groups, with five animals in each group. The pigs were inoculated intramuscularly with 10^7^ TCID_50_/animal of O/GD/CHA/2015 or O/CHA/7/2011, and the cattle were inoculated intradermally at multiple sites in the tongue with different doses of O/GD/CHA/2015 (1 × 10^7^ or 1 × 10^8^ TCID_50_/animal) or O/CHA/7/2011 (1 × 10^7^ or 1 × 10^8^ TCID_50_/animal) according to the standard protocol of the OIE. Clinical signs, including vesicular lesions and lameness, were monitored every day after viral challenge. Porcine samples of heparinized blood were collected at 1, 3, 5, 7, and 9 days postinoculation (dpi); bovine samples of heparinized blood were collected at 1, 3, 5, 7, 9, 15, 20, 25, and 30 dpi. The bovine samples of OPF were collected at 15, 20, 25, and 30 dpi. Clinical scores were determined by the vesicular lesion status of the challenged animals as previously described (26), with scores for the asymptomatic animals set at 0 and a maximum score of 5 for the infected animals. Lesions restricted to the inoculation site were not counted.

In the other two-animal experiments, different doses of PK-complete rescued virus (rO-WT) or rescued viruses with the 86-nt deletion (rO-DPKs86) or the 43-nt deletion (rO-DPKs43) in the PK region were used for viral challenge. After challenge, clinical signs were observed and recorded daily, and samples of heparinized blood and OPF were collected as described for the first experiment.

### Animal viral load quantification assay.

Total RNA was extracted from frozen heparinized blood and OPF samples during the animal experiments using an RNeasy kit (Qiagen, Hilden, Germany) according to the manufacturer’s protocol. FMDV viral RNA levels were measured by the qPCR method as previously described (48). Samples with threshold cycle (*C_T_*) values of ≤35 were considered positive.

### Data availability.

The genomic sequences of O/GD/CHA/2015 and O/CHA/7/2011 were submitted to GenBank under accession numbers KY234502 and KY234501, respectively.
